# Race/Ethnicity, Lipoproteins, and Antiretroviral Therapy

**DOI:** 10.1371/journal.pmed.0030098

**Published:** 2006-01-24

HIV-1 infection has become a chronic, manageable condition for patients who can get long-term access to highly active antiretroviral therapy (HAART). However, for some patients, HAART causes various metabolic complications, including the development of dyslipidemia. For example, some protease inhibitors (PI), a class of anti-HIV drugs, have been associated with elevated levels of cholesterol, triglyceride (TG), and low high-density lipoprotein cholesterol (HDL-c).

PI-based HAART has also been associated with increased risk for cardiovascular disease, which could, it has been suggested, lead to a future epidemic of cardiovascular disease in patients with HIV-1 treated with this regimen long term.

PIs are not the only anti-HIV drugs thought to play a role in dyslipidemia. Researchers suspect that non-nucleoside reverse transcriptase inhibitors (NNRTIs), a widely used class of antiretroviral, may also contribute to the condition. But the role of NNRTIs is less clear. The relationship between HIV-1 infection and its treatment is a complex one that we still do not completely understand, and it may have a profound impact on the future well-being of this population.

The features of PI-HAART-related dyslipidemia resemble familial combined hyperlipidemia (a lipid disorder that runs in families and which has a genetic basis), which suggests that lipoprotein gene variants might be linked to this condition. For example, apoC-III, a protein whose plasma levels are directly correlated with TGs in the general population, has also been implicated in regulating breakdown of triglyceride-rich lipoprotein. Several studies have established a complex interaction of genetic variation between apoC-III and apoA-I/C-III/A-IV/AV cluster with plasma TG levels. Two previous studies, restricted almost entirely to White individuals, reported a marked increase in plasma TG levels in patients with HIV-1 on PI-HAART regimens when they also had apoC-III and apoE gene variants.

Given the worldwide demographics of HIV-1 infection, the relationship between PI-HAART-related metabolic events and race/ethnicity could be important. The relationship between race/ethnicity and lipoproteins has been well described in the general population. Race/ethnicity has been considered a surrogate for environmental influences on lipids, although recent studies have demonstrated that genetic factors also account for important differences in plasma lipids across racial/ethnic groups. For example, studies have shown that some hepatic lipase single nucleotide polymorphisms (SNPs) present only in African Americans result in lower enzyme activity, and account for a proportion of the racial/ethnic differences in HDL-c levels.

The role of race/ethnicity in metabolic complications in individuals with HIV-1 has not really been considered. In a new study, Andrea Foulkes and colleagues examined the influence of racial/ethnic influences on plasma lipoproteins, and studied the genetic effects on lipids in patients with HIV-1. They did a cross-sectional analysis of race/ethnicity, apoC-III/apoA-I genotypes, and PI exposure on plasma lipids on 626 patients participating in several ongoing AIDS Clinical Trial Group studies. They found that race/ethnicity was a predictor of plasma lipids in patients with HIV-1 on HAART. Overall, Black patients on HAART had a less atherogenic lipid profile than White and Hispanic patients. The authors believe their finding was consistent with epidemiological data in non-HIV-1 populations, and could be of particular importance given the global spread of HIV-1 infection, especially in sub-Saharan Africa. They also found that in Hispanic, but not in White or Black, patients, apoC-III gene variants were associated with less dyslipidemia in patients exposed to PI-containing HAART.

Foulkes and colleagues say their findings are the first evidence, to their knowledge, for race/ethnicity–specific differences in both the occurrence of dyslipidemia on antiretroviral therapy (ART) and the influence of genetic factors on the prevalence of PI-related lipid abnormalities. But they stress that the findings do not imply that “race” was responsible for functional differences between gene variants. Rather, it is much more likely that differences relate to variation in the linkage disequilibrium (the inheritance pattern of blocks of DNA, or haplotypes, over time) in apoC-III, which they found to vary with race/ethnicity, or to the confounding influences of additional environmental and/or genetic factors.

Putting race/ethnicity aside, the authors rightly point out that any strategy that identifies individuals with HIV-1 at increased risk of ART-related metabolic complications will improve decision making for selecting appropriate ART regimens and preventive cardiovascular therapies, and ultimately will decrease the long-term cardiovascular risk of these patients. In addition, they emphasize that such strategies have to be assessed in studies that include adequate numbers of individuals within all racial/ethnic groups in order to determine their generalizability and utility in the worldwide population of patients with HIV-1.

